# BRET evidence that β2 adrenergic receptors do not oligomerize in cells

**DOI:** 10.1038/srep10166

**Published:** 2015-05-08

**Authors:** Tien-Hung Lan, Qiuju Liu, Chunman Li, Guangyu Wu, Jan Steyaert, Nevin A. Lambert

**Affiliations:** 1 Department of Pharmacology and Toxicology, Medical College of Georgia, Georgia Regents University, Augusta, GA 30912-2300 USA; 2 Structural Biology Brussels, Vrije Universiteit Brussel (VUB), and Structural Biology Research Center (VIB), Brussels, Belgium

## Abstract

Bioluminescence resonance energy transfer (BRET) is often used to study association of membrane proteins, and in particular oligomerization of G protein-coupled receptors (GPCRs). Oligomerization of class A GPCRs is controversial, in part because the methods used to study this question are not completely understood. Here we reconsider oligomerization of the class A β_2_ adrenergic receptor (β_2_AR), and reevaluate BRET titration as a method to study membrane protein association. Using inducible expression of the energy acceptor at multiple levels of donor expression we find that BRET between β_2_AR protomers is directly proportional to the density of the acceptor up to ~3,000 acceptors μm^−2^, and does not depend on the density of the donor or on the acceptor:donor (A:D) stoichiometry. In contrast, BRET between tightly-associating control proteins does not depend on the density of the acceptor, but does depend on the density of the donor and on the A:D ratio. We also find that the standard frameworks used to interpret BRET titration experiments rely on simplifying assumptions that are frequently invalid. These results suggest that β_2_ARs do not oligomerize in cells, and demonstrate a reliable method of assessing membrane protein association with BRET.

G protein-coupled receptors (GPCRs) are a large family of proteins with seven transmembrane domains that transduce signals across the cell surface after binding to hormones and neurotransmitters^1^. The largest subfamily of GPCRs is the rhodopsin-like class A, which includes the α and β adrenergic receptors for epinephrine and norepinephrine. The β_2_ adrenergic receptor (β_2_AR) has been widely used as a model GPCR, and was the first receptor after rhodopsin to have its primary and tertiary structures determined^2^,^3^. The β_2_AR was also one of the first GPCRs proposed to have a quaternary structure^4^. However, despite extensive study, the existence of class A GPCR dimers and oligomers as *bone fide* quaternary structures is uncertain. For example, several reports have appeared that both support^4^,^5^,^6^,^7^,^8^ and fail to support^9^,^10^,^11^,^12^,^13^ the existence of β_2_AR dimers and oligomers.

Some of the difficulty in reaching a consensus regarding class A GPCR oligomerization stems from the methods that must be used to study interactions between integral membrane proteins. Removing multispanning transmembrane proteins from their native membrane environment is likely to significantly alter their interactions, therefore biochemical methods that involve solubilization are not ideal for this purpose. For this reason biophysical methods that can be applied to receptors in living cells have been heavily relied upon to address this question. In particular, bioluminescence resonance energy transfer (BRET) has provided much of the evidence supporting class A GPCR oligomerization^14^,^15^,^16^,^17^,^18^,^19^, and is increasingly being used to assess oligomerization of other membrane proteins. BRET studies of oligomerization are most often based on the expectation that BRET between molecules that associate should be determined primarily by acceptor:donor stoichiometry (A:D), which determines the fraction of donors that are associated with acceptors^20^,^21^. Energy transfer between molecules that oligomerize is expected to reach a maximum (or “saturate”) when acceptors are in excess and all donors are associated with an acceptor (i.e. all are in D::A complexes). In contrast, BRET between molecules that do not associate should be determined primarily by the acceptor density ([A]), which determines the average distance between each donor and the nearest acceptors^21^,^22^. In most studies of this type (referred to as “titration”, “saturation” or “type-1” BRET assays) energy transfer is monitored as a function of the ratio A:D, which is varied by systematically increasing the relative expression of acceptors. The appearance of a non-linear or hyperbolic relationship between BRET and A:D is interpreted as evidence of oligomerization^9^,^17^,^18^,^23^.

Here we reevaluate this widely-used methodology, using oligomerization of the β_2_AR as a test case. We find that standard titration BRET protocols do not reliably distinguish associating and non-associating membrane proteins. Non-associating membrane proteins can generate a hyperbolic relationship between BRET and A:D, and associating membrane proteins may fail to do so. Using modified methods we find that BRET between β_2_AR protomers is directly proportional to acceptor expression, [A], and is not dependent on donor expression or the A:D ratio. These findings support the conclusion that these receptors do not oligomerize in cells.

## Results

### BRET between β_2_AR protomers with inducible expression of the acceptor

As part of a recent study we constructed a stable cell line that expresses the β_2_AR fused to the BRET acceptor venus (β_2_AR-V) under the control of a tetracycline-inducible promoter. This cell line expressed ~5 × 10^4^ radioligand binding sites per cell (~75 μm^−2^) without tetracycline induction, which increased 40-fold to ~2 × 10^6^ sites per cell (~3,000 μm^−2^) with tetracycline. It occurred to us that such cell lines might be useful for BRET studies that increase [A] in order to assess oligomerization. To our knowledge all such studies have used transient cotransfection of variable amounts of plasmid DNA to vary acceptor expression. This approach inevitably leads to acceptor expression that varies widely between cells and is difficult to measure accurately owing to incomplete transfection efficiency and background fluorescence. We reasoned that a cell line expressing the acceptor under the control of an inducible promoter would mitigate these problems. Accordingly, we induced β_2_AR-V expression with increasing concentrations of tetracycline, and transiently transfected the same cells with plasmid DNA encoding the β_2_AR fused to the BRET donor Rluc8 (β_2_AR-Rluc8). We expected that only a subpopulation of cells would express both β_2_AR-Rluc8 and β_2_AR-V, but since cells expressing only β_2_AR-V (i.e. cells that did not take up and express β_2_AR-Rluc8) would not contribute luminescence, these cells would not contribute to BRET measurements. This hybrid transfection protocol also provided a convenient means to independently manipulate expression of β_2_AR-V (by varying the tetracycline concentration), and β_2_AR-Rluc8 (by varying the time allowed for β_2_AR-Rluc8 accumulation after transient transfection or the amount of plasmid DNA).

**Figure 1:**
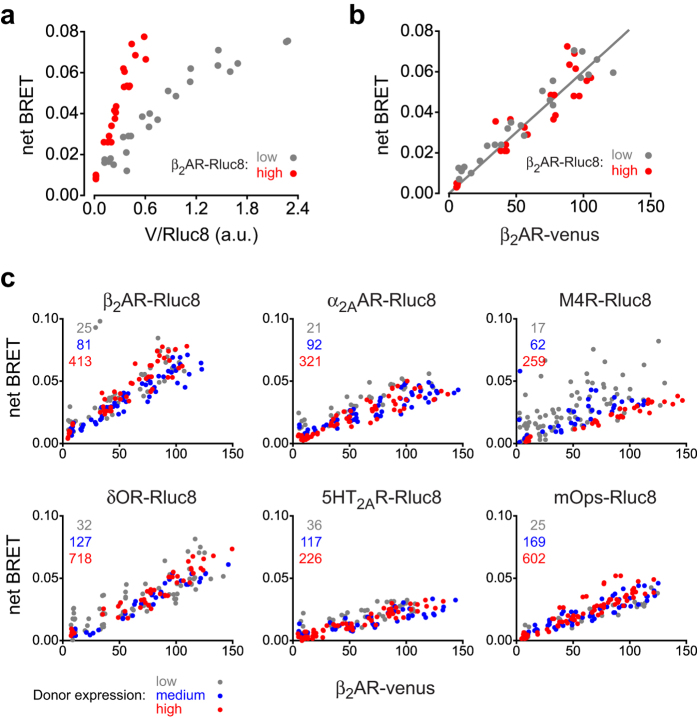
BRET between class A GPCRs and β_2_ adrenergic receptors depends on acceptor density, but not donor density or A:D. (**a**) net BRET plotted versus V/Rluc8 (arbitrary units, a.u.) for cells expressing β_2_AR-V under the control of a tetracycline inducible promoter that were transiently transfected with β_2_AR-Rluc8 for 12-48 hours to allow for different levels of donor expression. Each data point represents a single tetracycline concentration in a single experiment (*n* = 4). Low (*grey*) and high (*red*) levels of β_2_AR-Rluc8 expression averaged 53 × 10^3^ and 326 × 10^3^ photon counts well^−1^, respectively. (**b**) The same data as in panel **a** plotted versus β_2_AR-V expression (photon counts × 10^3^ well^−1^). A least squares fit to a straight line is superimposed. (**c**) experiments analogous to that shown in panel **b** with six different transiently transfected donors: β_2_ adrenergic receptors, α_2A_ adrenergic receptors, M4 muscarinic acetylcholine receptors, δ opioid receptors, 5HT_2A_ serotonin receptors, and mouse opsin. Donors were fused to *Renilla* luciferase (Rluc8), and were expressed for 12-48 hours to allow for accumulation of low (*grey*), medium (*blue*) and high (*red*) levels of expression (*n* = 2-7 at each donor expression level). Average expression of each donor at each level is indicated on each panel (photon counts × 10^3^ well^−1^).

In experiments of this type it has become conventional to plot net BRET (as an approximation of BRET efficiency, see Methods) against A:D with arbitrary units (a.u.), in this case β_2_AR-V fluorescence divided by β_2_AR-Rluc8 luminescence (V/Rluc8; Fig. 1a). As expected, increasing the expression of β_2_AR-V produced increasing BRET. However, BRET increased throughout the entire range of β_2_AR-V expression, meaning that energy transfer did not reach a maximum with increasing relative expression of the acceptor. This result was unexpected, as many studies of this type have documented saturating BRET between class A GPCRs, including β_2_ARs.

One possible explanation for our failure to observe saturating BRET with this protocol was that β_2_AR-V expression never reached the point where β_2_AR-V protomers significantly outnumbered β_2_AR-Rluc8 protomers. However, calibration with V-Rluc8 fusion proteins designed to minimize intramolecular BRET (see Methods) and flow cytometry (below) both indicated that β_2_AR-V expression exceeded β_2_AR-Rluc8 expression in the cells expressing both proteins by factors ranging from ~20:1 (with high β_2_AR-Rluc8) to ~500:1 (with low β_2_AR-Rluc8) at the highest levels of β_2_AR-V expression. In addition, transient transfection of β_2_AR-Rluc8 did not detectably increase the total number of β_2_AR receptors detected by radioligand binding in β_2_AR-V cells, suggesting that β_2_AR-Rluc8 protomers were a small minority. Moreover, it was evident that BRET versus V/Rluc8 relationships were distinct with different levels of β_2_AR-Rluc8 expression. BRET was higher at a given value of V/Rluc8 if β_2_AR-Rluc8 expression was higher (Fig. 1a), meaning that β_2_AR-V expression was also higher. This suggested that BRET between β_2_AR-Rluc8 and β_2_AR-V was not a fixed function of A:D *per se*, and that the absence of saturation in these experiments was not due to a stoichiometric excess of β_2_AR-Rluc8.

An alternative explanation for our failure to observe saturating BRET with increasing β_2_AR-V expression is that β_2_AR-Rluc8 and β_2_AR-V do not oligomerize, and BRET between these receptors is due to β_2_AR-Rluc8 and β_2_AR-V being in close proximity by chance (a.k.a. “bystander BRET”). Energy transfer between non-associated membrane proteins is expected to depend strictly on the acceptor density, [A], and not to depend on stoichiometry, A:D, or donor expression, [D]^21^. Indeed, BRET between β_2_AR-Rluc8 and β_2_AR-V appeared to be directly proportional to β_2_AR-V expression, as it was approximated well by a straight line. This relationship was the same across a wide range of β_2_AR-Rluc8 expression (Fig. 1b), therefore it did not depend on A:D or [D]. Since this result suggested that β_2_AR-Rluc8 and β_2_AR-V were not specifically associated, we predicted that we would be able to observe similar characteristics with other donors. Accordingly, we repeated this experiment with five additional class A GPCRs fused to Rluc8, and in every case observed BRET of similar magnitude that was proportional to β_2_AR-V expression across a wide range of donor expression (Fig. 1c).

**Figure 2:**
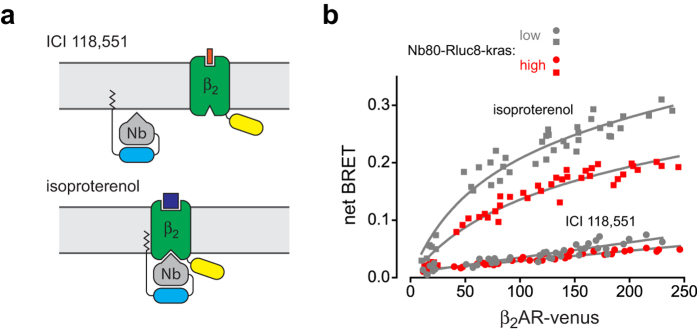
BRET is qualitatively different between associated and non-associated Nb80-Rluc8-kras and β_2_AR-V. (**a**) A schematic diagram illustrating the expected relationships between Nb80-Rluc8-kras and β_2_AR-V in the presence of the inverse agonist ICI 118,551 and the agonist isoproterenol. (**b**) BRET between Nb80-Rluc8-kras and β_2_AR-V plotted versus β_2_AR-V expression (photon counts × 10^3^ well^−1^) at low (*grey*) and high (*red*) levels of donor expression (30 × 10^3^ and 4,907 × 10^3^ photon counts well^-1^), in the presence of ICI 118,551 (1 μM) or isoproterenol (10 μM) (*n* = 6). Least squares fits to straight lines are superimposed on the ICI 118,551 data points, and least squares fits to a single site plus linear function (see Methods) are superimposed on the isoproterenol data points.

These results suggested that none of the class A GPCRs tested oligomerized with β_2_AR-V, and that we should be able to observe similar BRET between non-GPCR membrane proteins and β_2_AR-V. On the other hand, BRET between β_2_AR-V and a donor that actually associates with it should be qualitatively different. To test these predictions we constructed a membrane protein that could serve as both a negative (non-associating) and positive (associating) control in the same β_2_AR-V-expressing cell line. Nanobody 80 (Nb80) is a single-domain camelid antibody that was selected to bind and stabilize the active conformation of the β_2_AR, and has been used in cells as a sensor of β_2_AR activity^24^,^25^. A significant advantage of Nb80 for this purpose is that its affinity for inactive and active β_2_ARs has been measured *in vitro*, with equilibrium dissociation constants of ~1 μM and 3 nM, respectively^25^. We fused Nb80 to Rluc8 and a plasma membrane targeting peptide derived from K-Ras, and transfected this construct (Nb80-Rluc8-kras) into cells expressing β_2_AR-V. Cells were then exposed to the inverse agonist ICI 118,551 (1 μM) to maintain β_2_AR-V in the inactive state, or the agonist isoproterenol (10 μM) to promote formation of the active state (Fig. 2a). BRET between Nb80-Rluc8-kras and inactive β_2_AR-V closely resembled BRET between class A GPCRs and β_2_AR-V, both in terms of the magnitude of the BRET signal and the linear relationship between BRET and β_2_AR-V expression that did not depend on the level of Nb80-Rluc8-kras expression (Fig. 2b). In contrast, BRET between Nb80-Rluc8-kras and β_2_AR-V increased ~3-5 fold when the receptors were activated, and was no longer directly proportional to β_2_AR-V expression. An initial steep rise in BRET was followed by a more gradual increase as β_2_AR-V expression increased (Fig. 2b). Notably, BRET did not reach a clear maximum at high levels of β_2_AR-V expression, even though Nb80-Rluc8-kras and active β_2_AR-V presumably formed high affinity complexes. Moreover, BRET between Nb80-Rluc8-kras and active β_2_AR-V depended on both donor and acceptor expression, being more efficient at a given [A] when donor expression was lower (i.e. when A:D was greater). These results indicated that BRET versus β_2_AR-V relationships were qualitatively different for non-associating and associating membrane donors, in general agreement with theoretical predictions.

### Reevaluating titration BRET with transient cotransfection of the donor and acceptor

**Figure 3:**
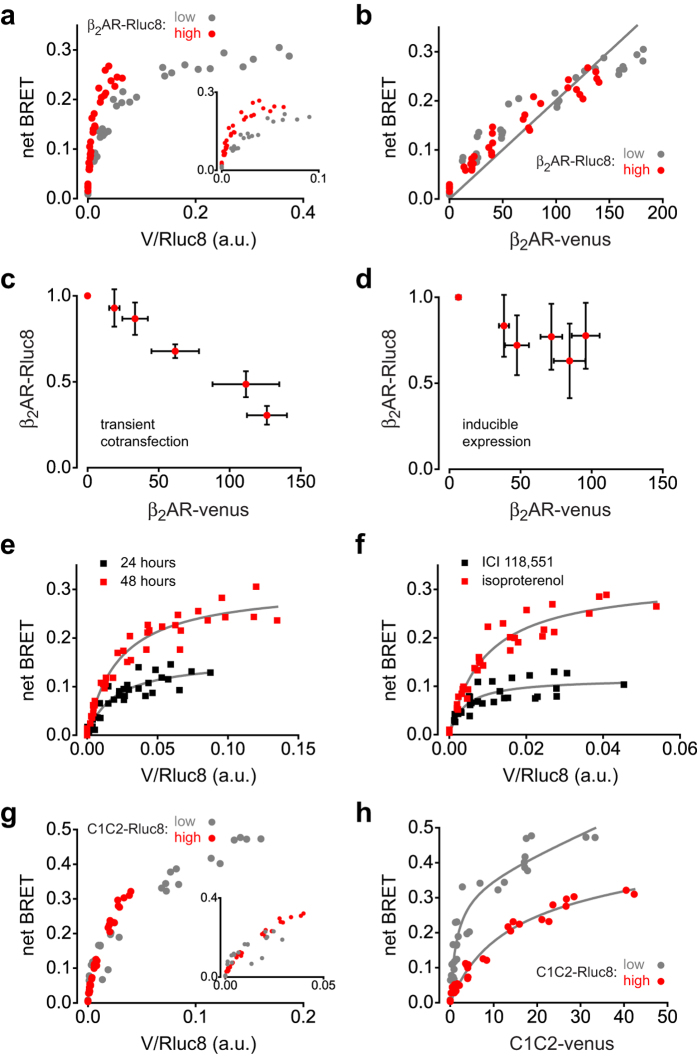
Decreasing donor expression confounds standard titration BRET after transient cotransfection. (**a**) net BRET plotted versus V/Rluc8 for cells expressing β_2_AR-Rluc8 and β_2_AR-V after transient cotransfection with two different concentrations of β_2_AR-Rluc8 DNA (0.05 and 0.5 μg well^−1^), each with the same range of β_2_AR-V DNA (0-2.5 μg well^−1^). Low (*grey*) and high (*red*) levels of β_2_AR-Rluc8 expression averaged 792 × 10^3^ and 3,340 × 10^3^ photon counts well^-1^ (*n* *=* *6*). (**b**) The same data as in panel **a** plotted versus β_2_AR-V expression (photon counts × 10^3^ well^-1^). A least squares fit to a straight line is superimposed. (**c**) Normalized β_2_AR-Rluc8 expression decreases as β_2_AR-V increases after transient contransfection. Data points represent the mean ± S.D. for the high donor data shown in panels **a** and **b**. (**d**) Normalized β_2_AR-Rluc8 expression also decreases as β_2_AR-V increases after tetracycline induction, although to a lesser extent. Data points represent the mean ± S.D. for the high donor data shown in Fig. 1a and b. (**e**) net BRET plotted versus V/Rluc8 for cells expressing β_2_AR-Rluc8 and β_2_AR-V 24 and 48 hours after identical transient cotransfection. Least squares fits to a single site binding function are superimposed. BRET_max_ was 0.157 after 24 hours, and 0.308 after 48 hours (*n* = 7). (**f**) net BRET plotted versus V/Rluc8 for cells expressing Nb80-Rluc8-kras and β_2_AR-V after transient cotransfection in the presence of ICI 118,551 (1 μM) or isoproterenol (10 μM). Least squares fits to a single site binding function are superimposed. BRET_50_ was 0.004 in ICI 118,551, and 0.009 in isoproterenol (*n* = 6). (**g**) net BRET plotted versus V/Rluc8 for cells expressing C1C2-Rluc8 and C1C2-V after transient cotransfection with two different concentrations of C1C2-Rluc8 DNA (0.05 and 0.5 μg well^−1^), each with the same range of C1C2-V DNA (0-2.5 μg well^−1^). Low (*grey*) and high (*red*) levels of C1C2-Rluc8 expression averaged 154 × 10^3^ and 843 × 10^3^ photon counts well^−1^ (*n* = 6). (**h**) The same data as in panel **g** plotted versus C1C2-V expression. Least squares fits to a single site plus linear function are superimposed.

Because these results differed from many previous reports that used BRET to study oligomerization of class A GPCRs we performed a series of experiments in an attempt to explain the discrepancies. The main methodological differences between our approach and that taken in previous studies were the method of acceptor expression (inducible expression as opposed to transient cotransfection) and the use of more than one level of donor expression. Accordingly, we mimicked the conditions of our first experiment by transiently cotransfecting cells with a range of plasmid DNA encoding β_2_AR-V (0 μg to 2.5 μg per well), and two different amounts of DNA encoding β_2_AR-Rluc8 (0.05 μg or 0.5 μg per well). We found that plots of BRET versus V/Rluc8 were again different with the two different levels of β_2_AR-Rluc8 expression (Fig. 3a, inset), but plots of BRET versus β_2_AR-V were superimposable (Fig. 3b), in both cases reproducing the results we obtained with inducible expression of β_2_AR-V. A notable difference, however, was that BRET approached a clear maximum level as V/Rluc8 increased (Fig. 3a), and also deviated slightly from linearity as a function of β_2_AR-V expression (Fig. 3b). These results are consistent with the vast majority of studies of GPCR oligomerization that have used similar methods^5^,^9^. However, it was evident that β_2_AR-Rluc8 expression decreased significantly when β_2_AR-V expression increased, even though the amount of β_2_AR-Rluc8 DNA was held constant in each experiment (Fig. 3c). With the highest level of β_2_AR-V cotransfection β_2_AR-Rluc8 expression was 31 ± 5% (*n* = 6) of that observed without β_2_AR-V cotransfection. Because decreasing donor expression could lead to saturating BRET versus A:D for non-associating molecules, the saturating BRET we observed between β_2_AR-Rluc8 and β_2_AR-V cannot be interpreted as evidence for association of these protomers. The decrease in β_2_AR-Rluc8 expression was less prominent after inducible expression of β_2_AR-V (Fig. 3d), consistent with the absence of clearly saturating BRET versus V/Rluc8 under these conditions (Fig. 1a). With the highest level of β_2_AR-V induction β_2_AR-Rluc8 expression was 78 ± 19% (*n* = 8) of that observed without β_2_AR-V induction. The reason for this difference between transient cotransfection and inducible expression is not immediately apparent, although lower absolute expression of β_2_AR-V after tetracycline induction (see below) may be a factor. Taken together our results with transient cotransfection, like our results with inducible expression, suggest that BRET between β_2_AR-Rluc8 and β_2_AR-V does not depend on A:D *per se*, but instead depends strictly on [A]. They also suggest that saturation of BRET versus A:D observed after transient cotransfection can be at least partly due to a decrease in donor expression as acceptor expression is increased.

Several additional observations made it clear that standard plots of BRET versus A:D after transient cotransfection could not safely be interpreted using the standard theoretical models^14^,^18^,^23^. For example, we regularly observed, as others have previously^9^, that the maximal BRET (BRET_max_) observed between β_2_AR-Rluc8 and β_2_AR-V increased over time after cotransfection, presumably due to increasing expression of β_2_AR-V (Fig. 3e). According to theory, BRET_max_ should be constant for a given oligomeric structure. Similarly, the A:D ratio at which BRET was 50% of the maximal level (BRET_50_) was higher for Nb80-Rluc8-kras with active β_2_AR-V than with inactive β_2_AR-V (Fig. 3f), even though the affinity of this donor for active β_2_AR-V is several orders of magnitude higher^25^. According to theory, BRET_50_ should report the change in affinity of this interaction in the opposite manner. These results underscore the conclusion that the hyperbolic shape of the BRET versus A:D relationship after transient cotransfection does not faithfully report oligomer structure or affinity.

Although the relationship between BRET and A:D at a single level of donor expression was not a reliable indicator of oligomerization, the constant relationship between BRET and [A] at different levels of [D] that we observed with β_2_ARs (Fig. 3b) suggested that varying both [D] and [A] systematically with cotransfection might allow discrimination of associating and non-associating membrane proteins. We therefore performed a cotransfection study with the channelrhodopsin chimera C1C2 using the same concentrations of plasmid DNA that were used with β_2_AR-Rluc8 and β_2_AR-V. This chimera is topologically similar to class A GPCRs, and forms constitutive, covalent dimers via N-terminal disulfide bonds^26^. BRET versus V/Rluc8 for C1C2-Rluc8 and C1C2-venus was similar with the two different levels of C1C2-Rluc8 expression in the range where the two relationships overlapped (Fig. 3g, inset). In contrast, BRET versus C1C2-venus expression relationships were dramatically different with the two different levels of C1C2-Rluc8 expression. BRET was much more efficient at a given [A] when donor expression was lower, and thus A:D was greater (Fig. 3h). Notably, we did not observe saturation of BRET between C1C2-Rluc8 and C1C2-venus, even though a large excess of the latter was present. Therefore, in contrast to BRET between β_2_AR protomers, BRET between C1C2 protomers depends on A:D, and is not simply proportional to [A].

### Cellular heterogeneity after transient cotransfection and inducible expression

**Figure 4:**
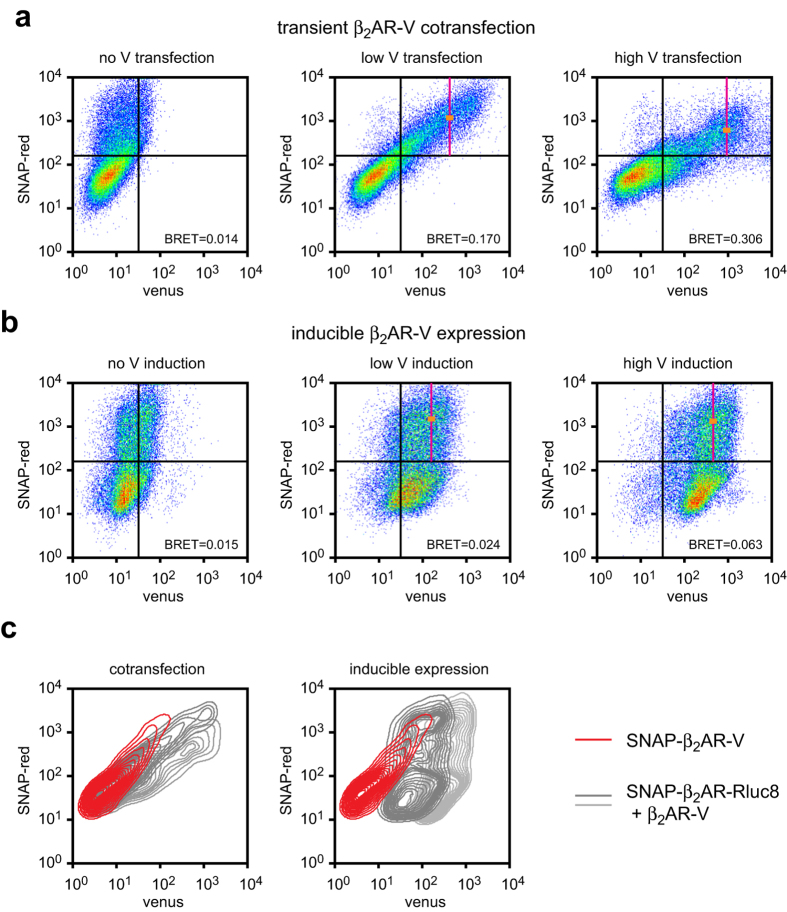
Donor and acceptor expression are positively correlated after transient contransfection. SNAP-tagged β_2_AR-Rluc8 (covalently labeled with SNAP-red) and β_2_AR-V expression were measured by flow cytometry. Gates were defined by staining untransfected cells with SNAP-red. Mean β_2_AR-V expression in coexpressing (upper right quadrant) cells is indicated by a vertical magenta line, and mean β_2_AR-Rluc8 expression in the same cells is indicated by an orange tick. BRET measured from each population of cells is indicated in each panel. (**a**) Transient cotransfection of constant SNAP-β_2_AR-Rluc8 (0.5 μg well^-1^) and increasing β_2_AR-V (0-2.5 μg well^−1^) produced positively correlated expression of the two proteins. Increasing the amount of β_2_AR-V DNA from 0.5 μg well^−1^ to 2.5 μg well^−1^ increased average β_2_AR-V expression in coexpressing cells from 426 arbitrary units (a.u.; CV = 1.54) to 936 a.u. (CV = 1.60), and decreased average β_2_AR-Rluc8 expression from 1,188 a.u. (CV = 1.13) to 612 a.u. (CV = 1.13). (**b**) Transient transfection of constant SNAP-β_2_AR-Rluc8 (0.5 μg well^−1^) with tetracycline induction of β_2_AR-V produced uncorrelated expression of the two proteins. Increasing the tetracycline concentration increased average β_2_AR-V expression in coexpressing cells from 162 a.u. (CV = 0.83) to 458 a.u. (CV = 0.82), and decreased average β_2_AR-Rluc8 expression from 1,502 a.u. (CV = 1.11) to 1,328 a.u. (CV = 1.13). (**c**) Cells expressing receptors with a single SNAP tag and a single venus (SNAP-β_2_AR-venus) were stained with SNAP-red and analyzed by flow cytometry (*red contour plots*). Superimposed (*grey*) contour plots from cells expressing SNAP-β_2_AR-Rluc8 and β_2_AR-V by both methods (the same cells as in panels **a** and **b**) show that most cells express more β_2_AR-V than SNAP-β_2_AR-Rluc8. This level of SNAP-β_2_AR-Rluc8 expression (48 hours after transfection) corresponds to the highest level of β_2_AR-Rluc8 expression in Fig. 1, and produced similar levels of luminescence. Each contour line represents 5% of the total cell population. Data are representative of 3 identical flow cytometry experiments.

A particularly notable difference between inducible and transient expression of β_2_AR-V was the absolute level of BRET observed, which reached approximately 3-fold higher levels after transient transfection. Since we found that BRET was directly proportional to β_2_AR-V expression, the higher BRET observed with transient transfection could have been due to higher levels β_2_AR-V expression in the cells producing BRET. To test this idea we performed flow cytometry to determine how absolute β_2_AR-V expression compared after inducible expression and transient cotransfection. For these experiments we replaced β_2_AR-Rluc8 with SNAP-β_2_AR-Rluc8. This construct included an N-terminal SNAP tag which was labeled with a red fluorophore, allowing measurement of both donor and acceptor expression in individual cells. We found that β_2_AR-V expression in cotransfected cells was highly variable (CV = 1.60) and was positively correlated with SNAP-β_2_AR-Rluc8 expression (Fig. 4a). A subpopulation of cells expressed high levels of β_2_AR-V after cotransfection with even the lowest amount of β_2_AR-V DNA, and this subpopulation also expressed high levels of SNAP-β_2_AR-Rluc8. BRET measured from the entire population of cells would arise disproportionately from this subpopulation. Increasing the amount of β_2_AR-V DNA increased average β_2_AR-V expression, but also depressed SNAP-β_2_AR-Rluc8 expression, consistent with what we observed with population measurements of Rluc8 luminescence. In contrast, β_2_AR-V expression was less variable after tetracycline induction (CV = 0.82), and was not correlated with SNAP-β_2_AR-Rluc8 expression (Fig. 4b). The highest levels of β_2_AR-V attained after transient cotransfection were several fold higher than the highest levels attained after inducible expression. Because SNAP-β_2_AR-Rluc8 and β_2_AR-V expression were not correlated after inducible expression of β_2_AR-V, BRET measured from these cells would not arise disproportionately from cells expressing higher levels of β_2_AR-V than the population average. High expression of β_2_AR-V that correlates with β_2_AR-Rluc8 expression thus provides an explanation for the high levels of BRET observed between these protomers after transient cotransfection.

### Intramolecular BRET and intermolecular BRET are additive

**Figure 5:**
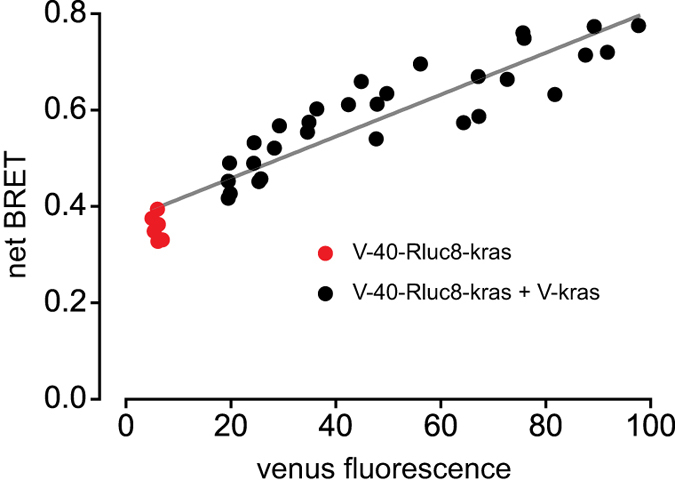
Intramolecular BRET does not preclude intermolecular BRET. Cells expressing V-40-Rluc8-kras were transiently cotransfected with an increasing amount of V-kras. Robust intramolecular BRET is increased further when additional membrane-bound acceptors are present (*n* = 6). A least squares fit to a straight line is superimposed.

Finally, we sought an explanation for our inability to reach a clearly saturating level of BRET as acceptor density increased with proteins that are known to associate tightly (Fig. 2b and Fig. 3h). Saturation is expected if it is assumed that BRET between associated donors and acceptors (e.g. within a D::A dimer) will be relatively efficient, and that energy transfer to “extra” non-associated acceptors (e.g. between D::A dimers or from D::A dimers to A::A dimers) will be negligible by comparison. We suspected that this assumption might not be valid, particularly if dimer structure is such that intramolecular BRET is inefficient. In this case additional energy transfer to extra acceptors should still be possible. To directly test this idea we fused venus to Rluc8 with an intervening 40 amino acid linker and a membrane tether (V-40-Rluc8-kras). This linker was chosen to allow intramolecular BRET of approximately the same magnitude as BRET observed between our control dimers (net BRET ~0.3-0.4). We then expressed extra acceptors in the form of membrane-tethered venus (V-kras), and observed additional BRET that was proportional to the level of V-kras expression. Indeed, despite robust BRET within V-40-Rluc8-kras “dimers”, it was possible to double net BRET by adding additional membrane-bound acceptors (Fig. 5). This result indicated that intramolecular BRET does not preclude intermolecular BRET, and therefore that oligomers should not be expected to produce saturating BRET. Accordingly, we found that BRET between our control associating proteins was well described by a function that included both saturating and linear components (fitted curves in Fig. 2b and Fig. 3h).

## Discussion

The starting point for the present study was the generation of an inducible cell line that expresses the prototypical class A β_2_ adrenergic receptor fused to the BRET acceptor venus. Using this cell line we unexpectedly observed that BRET between β_2_AR-Rluc8 and β_2_AR-V protomers is directly proportional to β_2_AR-V expression and is independent of β_2_AR-Rluc8 expression across a very wide range. Qualitatively similar BRET was observed with an assortment of class A donors, suggesting that none of the protomers studied associate as dimers or oligomers with β_2_AR-V. This conclusion was supported in the same cell line by an engineered control protein (Nb80-Rluc8-kras) that has vastly different affinities for inactive and active β_2_ARs. These findings suggest that β_2_ARs do not form a significant number of dimers or oligomers under these conditions.

These observations prompted us to reevaluate quantitative BRET using the cotransfection methods that have become standard practice. One of the main conclusions of this study is that the shape of the relationship between BRET and the A:D ratio is potentially misleading as an indicator of membrane protein oligomerization, particularly after transient cotransfection of donors and acceptors. Donor expression often decreases substantially as acceptor expression increases, even when the amount of DNA encoding the donor is kept constant. When this occurs it is possible to observe a hyperbolic or saturating relationship between BRET and A:D for non-associating membrane proteins. Although most studies of this type keep the amount of DNA encoding the donor constant, actual donor expression is not routinely reported. Therefore, the extent to which decreased donor expression contributed to the appearance of saturating BRET observed in previous studies of membrane proteins is difficult to ascertain. Even if it were possible to fix donor expression across a range of acceptor expression, expressing BRET as a function of A:D would not be advantageous, as the resulting plot would then be identical to a plot of BRET versus [A]. Combining [A] and [D] as an A:D ratio introduces unnecessary ambiguity, as it is not possible to distinguish BRET changes that occur due to changes in stoichiometry from those that occur due to changes in acceptor density.

A related conclusion is that systematic variation of [A] is most useful if it is combined with systematic variation of [D]. Direct assessment of energy transfer as a function of [A] at two or more levels of [D] reliably discriminated associating and non-associating membrane proteins. If BRET is similar at a given value of [A] with different values of [D] (e.g. Fig. 1b), this suggests that the donor and acceptor are not associated. The same conclusion can be drawn if BRET varies widely at a given value of A:D (e.g. Fig. 1a, Fig. 3e), which is the principle that underlies so-called BRET dilution or “type-2” quantitative BRET assays. However, it should be noted that energy transfer between associating molecules may not be constant at a given A:D due to energy transfer between oligomers, and therefore BRET versus A:D curves may not be identical even for associating molecules. These conclusions are virtually identical to those drawn by Szalai *et al.*, who recently presented an extensive reevaluation of quantitative saturation BRET with transient cotransfection^27^.

One of the simplifying assumptions underlying standard titration BRET analysis is that BRET between associating molecules should reach a maximum when the acceptor greatly outnumbers the donor. This is expected to be the case both for constitutive (permanent) oligomers as well as for transient oligomers, although in the latter case maximum energy transfer is expected to occur only when the monomer-oligomer equilibrium heavily favors the oligomeric state. We found that this simplifying assumption is frequently violated. The model proposed by Veatch and Stryer relating energy transfer to A:D and oligomerization state assumed that there were no interactions between oligomers, that all molecules oligomerized, and that intraoligomer energy transfer was 100% efficient^20^. These assumptions were carried over into derivatives of this model^5^,^9^,^16^,^17^,^18^,^23^, and the possibility that non-associated acceptors might significantly contribute to energy transfer even when all donors are associated with acceptors has generally been discounted. As a consequence, it is commonly expected that BRET between associating molecules will saturate or reach a maximum value (BRET_max_) with increasing [A] when A:D>1. However, we were unable to observe clearly saturating BRET versus [A] even between tightly associating control proteins. We were able to show that intramolecular and intermolecular BRET could be additive and of similar magnitude, thus providing an explanation for non-saturating BRET between associating proteins. In retrospect it is not surprising that BRET generated by dimers and oligomers may not reach a maximum value as acceptors outnumber donors. It is clear that BRET within a D::A complex can be far from 100% efficient, and that BRET between non-associated membrane proteins can be robust, and therefore that additional acceptors might substantially increase overall energy transfer^28^. Indeed, it is possible to envision a situation where BRET within D::A complexes does not occur while BRET between complexes does occur^29^. Therefore, it is reasonable to expect that BRET generated by membrane associated oligomers will include both intraoligmer and interoligomer components, and that the relative magnitude of each component will be determined by several factors. These would include the A:D ratio, acceptor density, affinity between protomers, structural features of the oligomers and their labels, and the distance of closest approach of donor and acceptor labels. The relative contributions of intraoligomer and interoligomer energy transfer may be difficult to predict, as recently demonstrated by numerical simulation^29^. Analyses of this type rely on several additional simplifying assumptions, many of which are not easily validated. These include assumptions regarding the efficiency of donor and acceptor maturation and function, and possible effects of donor and acceptor moieties on the association of fused proteins.

An often overlooked limitation of BRET studies is the technical necessity of making ensemble measurements from large populations of cells. Quantitative BRET studies have made the implicit assumption that ensemble measurements of BRET, fluorescence and luminescence are made from a homogeneous cell population, or at least that BRET originates from a population that is fairly represented by ensemble measurements. However, with standard transient cotransfection we found that donor and acceptor expression were positively correlated within each population of cotransfected cells. This means that the cells that express the most donor, and therefore contribute the most to ensemble BRET signals, also express more acceptor than the population average. BRET at “low” levels of acceptor expression will be dominated by a subpopulation of cells that express more acceptor than indicated by the ensemble average. Previous quantitative BRET studies have related receptor number (determined by radioligand binding) to ensemble fluorescence measurements, but have not considered heterogeneous expression. This error can be compounded if ensemble measurements are not corrected for incomplete transfection efficiency. These factors should be taken into account before concluding that BRET arises from cells that express a given level of donor or acceptor (e.g. native levels). Ensemble measurements that are not representative of the cells that produce BRET could produce significant deviation from simple theoretical frameworks, and our results emphasize that caution should be exercised whenever data obtained from potentially heterogeneous cell populations are interpreted using quantitative models.

Although it is more labor intensive, fluorescence resonance energy transfer (FRET) imaging can circumvent these problems, and can provide measurements of energy transfer, donor density and acceptor density from single cells and vesicles. The hybrid transfection method described here still relies on ensemble measurements, but holds some advantages for quantitative BRET studies. Most importantly, donor and acceptor expression are uncoupled and are not positively correlated, therefore ensemble measurements more faithfully represent the cells that generate BRET signals. In addition, acceptor expression is relatively uniform and easier to measure using standard spectrofluorimetry, and receptor numbers calculated using radioligand binding need not be corrected for incomplete transfection efficiency. Decreases in donor expression that accompany increases in acceptor expression are less pronounced than those that occur after transient cotransfection. Using this protocol we determined that BRET between β_2_ARs increases as a linear function of acceptor density across levels of expression between ~75 and ~3,000 acceptors per square micron, and does not depend on donor expression or A:D. BRET between randomly distributed donors and acceptors with a Förster distance (R_0_) of 5.5 nm (the estimated value for Rluc8 and venus)^30^ would be expected to increase as a linear function of reduced acceptor density within this range (<0.1 acceptors per R_0_^2^)^22^. BRET was virtually undetectable at the lowest levels of acceptor expression (~75 receptors μm^−2^) despite the high sensitivity of this method. This result is consistent with that of Kawano, *et al.*^10^, who recently were unable to detect FRET between β_2_ARs expressed at 66 receptors μm^−2^ unless they were first crosslinked. Similarly, a recent study used purified β_2_ARs reconstituted into proteoliposomes to estimate a dissociation constant *K*_d_ of 517 μm^−2^, which is consistent with the absence of substantial energy transfer at low expression levels in cells^31^. On the other hand, this value implies that a significant fraction of β_2_AR protomers should be associated with each other at the highest levels of expression achieved in our study (~6-fold greater than the calculated *K*_d_). One possible explanation for our failure to observe evidence of oligomerization at high expression levels is the presence of factors in native cell membranes that dramatically destabilize lateral interactions between transmembrane proteins^32^,^33^.

In summary, the results of the present study highlight some of the deficiencies of quantitative BRET analyses that rely on transient cotransfection of a single level of donor DNA and evaluation of BRET as a function of the A:D ratio. Proteins that do not associate can produce BRET that saturates as A:D increases due to decreases in donor expression. Proteins that do associate can produce BRET that does not saturate due to additional energy transfer to non-associated acceptors. Our findings with BRET between β_2_ARs do not fully explain previous reports of β_2_AR oligomerization^4^,^5^,^7^,^8^, but are nonetheless most consistent with the suggestion that these receptors do not readily form dimers or oligomers in cells^9^,^10^,^11^,^12^,^13^.

## Methods

### Plasmid DNA constructs

A plasmid encoding Rluc8 was provided by Dr. Sanjiv Sam Gambhir (Stanford University, Palo Alto, CA). SNAP-β_2_AR was obtained from New England Biolabs (Ipswich, MA). V-TRAF-Rluc8 and V-40-Rluc8 were provided by Dr. Stephen Ikeda (NIAAA, Rockville, MD). Plasmids encoding GPCRs were obtained from the Missouri S&T cDNA Resource Center (Rolla, MO) with the exception of mouse opsin (mOps), which was provided by Dr. Marina Gorbatyuk (University of Florida, Gainesville, FL). Rluc8 and venus fusions were made to the C-terminus of each receptor with either a GSGG linker or without an intervening linker. Nb80-EGFP was provided by Dr. Mark von Zastrow (University of California, San Francisco, CA). All fusion constructs were made using an adaptation of the QuikChange (Agilent Technologies, Santa Clara, CA) mutagenesis protocol and were verified by automated sequencing.

### Transient cotransfection of HEK 293 cells

HEK 293 cells (ATCC) were propagated in plastic flasks and on 6-well plates according to the supplier’s protocol. Cells were transiently transfected in growth medium using linear polyethyleneimine (PEI; MW 25,000; Polysciences Inc., Warrington, PA) at an N/P ratio of 20, and were used for experiments 12-48 hours later. Up to 3 μg of plasmid DNA was transfected in each well of a 6-well plate. In each case the total amount of DNA was kept constant by adding empty vector.

### Generation and transfection of Flp-In T-REx 293 cells

Flp-In T-REx HEK 293 cells (Invitrogen, Carlsbad, CA) were maintained in growth medium, supplemented with 15 μg ml^−1^ blasticidin and 100 μg ml^–1^ zeocin, and were transfected with a 1:9 ratio of β_2_AR-V/FRT/TO or mem-FRB-V/FRT/TO and pOG44 in growth medium using linear polyethyleneimine. 24 hours after transfection, cells were reseeded in medium supplemented with 100 μg ml^–1^ hygromycin B and 15 μg ml^−1^ blasticidin but without zeocin to select stably transfected cells. Positive cell colonies were collected as a pool and screened by fluorescence microscopy. For BRET experiments these cells were exposed to 0-0.1 μg ml^–1^ tetracycline to induce β_2_AR-V expression, and transfected with a BRET donor using linear PEI 0-36 hours later. BRET measurements were made 48 hours after tetracycline induction (12-48 hours after donor transfection). Due to <100% transient transfection efficiency not all cells expressed BRET donors in these experiments. However, untransfected cells that express only the acceptor do not contribute to population BRET measurements.

### Radioligand binding

Flp-In T-REx β_2_AR-V cells were seeded onto 24-well plates at 2 × 10^5^ cells per well. Expression of β_2_AR-V was induced by incubating with tetracycline (0-0.1 μg ml^−1^) for 24 hours. After induction cells were incubated in MEM containing 30 nM [^3^H]-CGP12177 for 90 minutes at room temperature. Cells were washed twice with ice cold MEM, and surface-bound ligand was extracted with 0.5 ml of 1 M NaOH for 2 hours. Radioactivity was counted by liquid scintillation counting in 3.5 ml of Ecoscint A (National Diagnostics, Atlanta, GA). Nonspecific binding was determined in the presence of 20 μM alprenolol. Binding sites per cell was calculated by comparing the specific radioactivity recovered from each well to the radioactivity of a known input (0.6 pmol), and dividing by the number of cells in each well. Receptor density was calculated using the surface area of HEK 293 cells (670 μm^2^) measured in a previous study^34^.

### BRET

Cells were washed with PBS, harvested by trituration, and transferred to opaque 96-well plates. Fluorescence and luminescence measurements were made using a Mithras LB940 photon-counting plate reader (Berthold Technologies GmbH, Bad Wildbad, Germany). Fluorescence emission was measured at 520-545 nm after excitation at 485 nm. Background fluorescence from untransfected cells was subtracted. For BRET coelenterazine h (5 μM; Dalton Pharma, Toronto, Ontario, Canada) was added to all wells immediately prior to making measurements. Raw BRET signals were calculated as the emission intensity at 520-545 nm divided by the emission intensity at 475-495 nm. Net BRET was this ratio minus the same ratio measured from cells expressing only the BRET donor. An important limitation of BRET in live cells is that actual efficiency of energy transfer between the oxidized luciferase substrate (coelenteramide) and the acceptor cannot be measured. The relationship between net BRET and BRET efficiency is not clear, but for the purposes of this study (and all previous quantitative BRET studies) net BRET is assumed to be proportional BRET efficiency. All experiments were performed at room temperature (~25° C).

### Flow cytometry

Cells expressing SNAP-tagged receptors were labeled with 80 nM SNAP-red (Cisbio, Codolet, France) for 2 hours at 37° C in complete growth medium. Cells were washed three times in PBS, harvested by trituration, and fixed with 4% paraformaldehyde in PBS. Flow cytometry was performed using a FACSCalibur (BD Biosciences, San Jose, CA) and 488 nm and 633 nm excitation lasers. Venus was detected in FL1 (530/30 nm) and SNAP-red was detected in FL4 (661/16 nm). Gates for background were determined using untransfected cells stained with SNAP-red. Identical excitation and detection amplifier settings were used across all experiments, and data were analyzed with FlowJo (Tree Star, Ashland, OR).

### Estimation of A:D

To estimate A:D with population measurements we constructed fusion proteins with a fixed 1:1 stoichiometry of venus and Rluc8 and intervening linkers designed to minimize intramolecular BRET. In one construct the linker was a transmembrane domain (V-TM-Rluc8) and in another the linker was a 230 amino acid fragment of TNF receptor-associated factor 2 (V-TRAF-Rluc8). Results from the two constructs were similar and were pooled. Fluorescence (F) and luminescence (L) readings from cells transiently transfected with these constructs were plotted against each other and fitted by linear regression, yielding an F/L ratio of 0.005 for a 1:1 A:D (V:Rluc8) ratio. This value was corrected to 0.015 for 33% donor transfection efficiency (estimated using flow cytometry) when BRET donors were transfected into stable β_2_AR-V-expressing cells. At the highest level of β_2_AR-Rluc8 expression (413 × 10^3^ photon counts well^−1^ in Fig. 1c) A:D ranged from 0.9:1 (with no β_2_AR-V induction) to 23:1 (with full β_2_AR-V induction). At the lowest level of β_2_AR-Rluc8 expression the corresponding ratios were 39:1 and 598:1.

To indicate A:D with flow cytometry we constructed a fusion protein with an extracellular SNAP tag, an intracellular venus and an intervening β_2_AR (SNAP-β_2_AR-V). Cells expressing this construct were labeled with SNAP-red and processed for flow cytometry under the same conditions as cells expressing SNAP-β_2_AR-Rluc8 and β_2_AR-V. By comparison with cells expressing SNAP-β_2_AR-V it was evident that most cotransfected and induced cells expressed more acceptor than donor (A:D > 1), even at the lowest levels of β_2_AR-V transfection or induction (Fig. 4c).

### Curve fitting

Non-linear least squares fitting was performed using a Levenberg–Marquardt algorithm to either a single site binding function, BRET = [(BRET_max_ × A:D)/(BRET_50_ + A:D)], for BRET versus A:D (Fig. 3e,f), where BRET_max_ is the maximal BRET observed, and BRET_50_ is the value of A:D at 50% of BRET_max_. BRET versus [A] (Fig. 2b, 3h) was fitted to a single site plus linear function, BRET = [(BRET_max_ × [A])/(BRET_50_ + [A])] + m[A], where BRET_max_ is the BRET value observed within a D::A complex, BRET_50_ is the value of [A] where half of all donors are associated with acceptors, and m is the slope of intermolecular BRET versus [A].

## Additional Information

**How to cite this article**: Lan, T.-H. *et al*. BRET evidence that β2 adrenergic receptors do not oligomerize in cells. *Sci. Rep.* **5**, 10166; doi: 10.1038/srep10166 (2015).
